# Psychological Distress During the Retirement Transition and the Role of Psychosocial Working Conditions and Social Living Environment

**DOI:** 10.1093/geronb/gbab054

**Published:** 2021-08-16

**Authors:** Mirkka Lahdenperä, Marianna Virtanen, Saana Myllyntausta, Jaana Pentti, Jussi Vahtera, Sari Stenholm

**Affiliations:** 1 Department of Public Health, University of Turku and Turku University Hospital, Finland; 2 Centre for Population Health Research, University of Turku and Turku University Hospital, Finland; 3 School of Educational Sciences and Psychology, University of Eastern Finland, Joensuu, Finland; 4 Clinicum, Faculty of Medicine, University of Helsinki, Finland

**Keywords:** Life event, Mental well-being, Pensioners, Social capital, Transitional period

## Abstract

**Objectives:**

Mental health is determined by social, biological, and cultural factors and is sensitive to life transitions. We examine how psychosocial working conditions, social living environment, and cumulative risk factors are associated with mental health changes during the retirement transition.

**Method:**

We use data from the Finnish Retirement and Aging study on public sector employees (*n* = 3,338) retiring between 2014 and 2019 in Finland. Psychological distress was measured with the General Health Questionnaire annually before and after retirement and psychosocial working conditions, social living environment, and accumulation of risk factors at the study wave prior to retirement.

**Results:**

Psychological distress decreased during the retirement transition, but the magnitude of the change was dependent on the contexts individuals retire from. Psychological distress was higher among those from poorer psychosocial working conditions (high job demands, low decision authority, job strain), poorer social living environment (low neighborhood social cohesion, small social network), and more cumulative risk factors (work/social/both). During the retirement transition, greatest reductions in psychological distress were observed among those with poorer conditions (work: absolute and relative changes, *p* [Group × Time interactions] < .05; social living environment and cumulative risk factors: absolute changes, *p* [Group × Time interactions] < .05).

**Discussion:**

Psychosocial work-related stressors lead to quick recovery during the retirement transition but the social and cumulative stressors have longer-term prevailing effects on psychological distress. More studies are urged incorporating exposures across multiple levels or contexts to clarify the determinants of mental health during the retirement transition and more generally at older ages.

Mental disorders are one of the leading causes of ill-health and disability, increasing the risk of chronic physical conditions and mortality (Lawrence et al., 2010; Scott et al., 2016). Mental health is determined by a range of socioeconomic, biological, and cultural factors and is sensitive to major life changes and transitions. Retirement is an important transitional period in late adulthood, traditionally considered as a stressful event, having negative consequences on mental health due to changes in established routines, loss of work-related roles and activities, and reduced income levels. However, there is accumulating evidence of retirement having positive effects on mental health (e.g., Oshio & Kan, 2017; Van Der Heide et al., 2013; Westerlund et al., 2010), potentially as a consequence of relief from stressful work, increased leisure time, increased physical activity (Ding et al., 2016; Oshio & Kan, 2017; Stenholm et al., 2016), longer sleep duration (Myllyntausta et al., 2017), and fewer sleep difficulties (Myllyntausta et al., 2018; Vahtera et al., 2009).

The associations between retirement and mental health may depend on the context individuals retire from (Fleischmann et al., 2020; Jokela et al., 2010; Westerlund et al., 2010; Wheaton, 1990). This is in line with the life-course perspective theory, according to which the experience of life transitions and subsequent developmental trajectories is dependent on the circumstances under which the transition occurs (Wang, 2007). Such circumstances include both conditions at work and private life. For example, high job strain is associated with adverse health outcomes (Habibi et al., 2015) and increased risk of mental health problems (Harvey et al., 2018). Retirement from a high-stress job as indicated by high job strain, high psychosocial job demands, low decision authority, and low social support has been associated with a decrease in these symptoms (Fleischmann et al., 2020; Wang, 2007; Westerlund et al., 2010; Wheaton, 1990), although the effect may be relatively short term, occurring within 3 years after retirement only (Fleischmann et al., 2020). A similar association between depressive symptoms, retirement, and previous psychosocial working conditions was found among retirees in Sweden, by using trajectory analysis approach (Åhlin et al., 2020). However, in their study, improvement in mental health was observed only for a small group of retirees with poor work conditions (e.g., higher job strain and lower workplace social support), while another group of people who also had poor psychosocial working conditions showed persistent depressive symptoms (Åhlin et al., 2020). This suggests that the effects of retirement may vary between individuals, and factors not related to work may explain part of the differences. In addition, the previous studies highlight that changes may occur relatively soon after retirement. Therefore, further research with shorter measurement intervals is warranted to understand how quickly the improvements in different measures of mental health occur after retirement, and whether other contexts than working conditions are involved in this association.

Social contexts are important determinants of the subsequent adjustment processes during life transitions (Wang, 2007). Social characteristics have been shown to be closely linked to several health behaviors and outcomes, including mental health (Alegría et al., 2018; Lund et al., 2018) and older adults already facing psychosocial stressors may be psychologically more vulnerable to adverse environmental conditions (Evans, 2003). Particularly, poor social living environment at the individual level (e.g., loneliness [Bulloch et al., 2017] and lacking social network [Degnan et al., 2018]) as well as at the neighborhood level (e.g., low social cohesion [De Silva et al., 2005] and high socioeconomic disadvantage [Kivimäki et al., 2020]) increase the risk of poor mental health. The importance of the neighborhood context may become greater as more time is spent in the neighborhood after retirement. Although the studies of neighborhood-level characteristics on mental health during retirement are rare, a recent study found that living in an area with significant neighborhood disorder and lack of social cohesion increased the chance of developing depression and the effects became stronger after retirement (Baranyi et al., 2020). Thus, an important research direction is the accumulation of adverse social conditions at individual and neighborhood level and mental health (Alegría et al., 2018).

Support from an individual’s social living environment may alleviate the effects of stressful life events, such as the retirement transition, on mental health but has a minor role in health for those without stressful life events (Cohen, 2004). Although the literature on social support and mental health is extensive, the role of social support during the retirement transition is poorly understood and the findings are mixed. Supporting the social buffering hypothesis, social support from friends (Kail & Carr, 2020) or spouse (Dave et al., 2008; Wang, 2007) has been found to mitigate the adverse effects of retirement on mental health in some studies. Contradictory to the social buffering hypothesis, retirement had the most beneficial health effects for those in worse social contexts, as indicated by a low socioeconomic status (Kolodziej & García-Gómez, 2019; Schuring et al., 2015; Westerlund et al., 2009). To identify whether different neighborhood and individual-level social contexts are associated differently with changes in mental health during the retirement transition, more studies are needed in different populations and pension systems.

This study builds on repeated measurements of psychological distress before and after retirement on Finnish public sector employees retiring between 2014 and 2019 with various occupations. The aim of this study was to investigate short-term changes in psychological distress during the retirement transition and whether these changes were associated with psychosocial working conditions and social living environment prior to retirement. Because physical health may be linked to mental health and retirement timing, we take disease status into account in the analysis (e.g., Vyas & Okereke, 2020). We build our hypotheses on life course perspective, social buffering hypothesis, and previous empirical findings. It has been found that the study participants sleep better, are physically more active, and have better self-rated health after retirement (Myllyntausta et al., 2017, 2018; Stenholm et al., 2016, 2020), thus we expect psychological distress to decrease during the retirement transition. We hypothesize, first, that people from poorer psychosocial working conditions have greater decreases in psychological distress during the retirement transition. Second, we hypothesize that people with greater social support at the individual level (being married, larger social network size) or neighborhood level (lower socioeconomic disadvantage, higher social cohesion) have greater decreases in psychological distress during the retirement transition. Third, we hypothesize that people with a higher number of accumulated work-related risk factors/ a lower number of accumulated social risk factors have greater decreases in psychological distress during the retirement transition, but when the risk factors accumulate in both contexts simultaneously, the retirement transition will be associated with a smaller resolution of risk factors only and, consequently, fewer improvements in mental health.

## Methods

### Study Population

The study population consists of participants of the Finnish Retirement and Aging (FIREA) study (Leskinen et al., 2018). The FIREA study was conducted in line with the Declaration of Helsinki and was approved by the Ethics Committee of Hospital District of Southwest Finland (84/1801/2014). The FIREA study cohort includes all public sector employees whose estimated retirement dates were between 2014 and 2019 and who were working in year 2012 in one of the 27 municipalities in Southwest Finland or in the nine selected cities or five hospital districts around Finland. Information on individual estimated retirement date was obtained from the pension insurance institute Keva. Study participants were first contacted 18 months prior to their estimated retirement date by sending a questionnaire, which was then sent annually, at least four times in total. The majority of the FIREA study participants retired based on their age and not due to disease or disability to work. By the end of year 2019, 6,783 study participants had responded to at least one questionnaire and 3,426 both prior to (−1) and after (+1) the actual self-reported retirement date. The study population for the current study was restricted to 3,338 participants, who had information on psychological distress at the study waves immediately before (−1) and after (+1) retirement. However, the final sample sizes varied slightly depending on the availability of information of prevailing working conditions variables and/or social living environment variables and the adjusted variables in each model.

### Psychological Distress

We used the 12-item General Health Questionnaire to measure symptoms of common mental health problems before and after retirement (Goldberg, 1972). The validated measure includes 12 questions regarding how well an individual feels like coping with difficulties, self-confidence, happiness, and depression (Supplementary Table 1). FIREA participants were asked to rate the extent to which they had recently experienced any of the 12 symptoms, using a 4-point Likert scale. The total score ranged from 0 to 12 and was used as a continuous outcome for this study.

### Psychosocial Working Conditions

The participants reported their psychological job demands, skill discretion, and decision authority, at study wave −1 prior to retirement by using Karasek’s Job Content Questionnaire (Karasek et al., 1998; Supplementary Table 1). The Cronbach’s α were 0.85, 0.75, and 0.78 for job demands, skill discretion, and decision authority, respectively. The mean score for job demands, skill discretion, and decision authority was 3.20 ± 0.85, 3.72 ± 0.62, and 3.54 ± 0.88, respectively. Job demands, skill discretion, and decision authority measures were divided to categories low/middle/high based on the tertiles (Joensuu et al., 2014). In addition, a measure indicating job strain was created combining job demands (median split) and job control (median split). High demands and low control were defined as job strain, while the other three combinations were defined as no job strain.

### Social Living Environment

Social living environment was measured by four indicators, including neighborhood-level as well as individual-level measures, reported at study wave −1. First, a summary *z* score representing neighborhood socioeconomic disadvantage at each participant’s residential area was obtained from Statistics Finland and is based on the level of education, the unemployment rate, and annual income in 250-m × 250-m grids (Kivimäki et al., 2020). The summary score was divided into three categories, low, middle, and high, by using values −0.5 and +0.5 as cutoffs. Second, neighborhood social cohesion (Sampson et al., 1997) measured trusting relationships of residents in a neighborhood (Supplementary Table 1). Cronbach’s alpha was 0.86 for the present sample. The values were divided into three categories, low, middle, and high, by using integers of tertiles as cutoffs. Third, social network size was assessed using the social convoy model (Antonucci, 1986; Supplementary Table 1). The model is based on a set of three concentric circles representing different levels of closeness to the respondent. Overall social network size was determined as a total number of network members in all three circles and was categorized as small (0–10 members), middle (11–20 members), or large (≥21 members; Kauppi et al., 2018). Fourth, individual marital status was coded as a two-categorical variable, married/cohabiting or living alone (including also widows/widowers and divorced).

### Cumulative Risk Factors

Cumulative risk factors were first calculated separately for poor psychosocial working conditions and social living environment. The poorest working conditions were coded as having a risk status (i.e., high job demand, low skill discretion, low decision authority, job strain). A summary score indicating the number of risk statuses was then calculated, dividing individuals into having no risks, one risk, and two or more risks in working conditions. Similarly, potentially adverse social living environments were coded as having a risk status (i.e., high socioeconomic disadvantage, low neighborhood social cohesion, small social network size, living alone) and individuals were divided into having no risks, one risk, and two or more risks in social environment. Finally, a combined cumulative risk factor (no risks, one, two, three, four or more risks) was calculated merging psychosocial working conditions and social environment indicators with eight potential risk factors.

### Background Factors

Information from the study wave −1, prior to retirement, was used to define all background variables and they were chosen because these factors may potentially associate with psychological distress levels during the retirement transition. Age, gender, and occupational status were obtained from the pension insurance institute for the municipal sector in Finland (Keva). Occupational status was categorized into three groups according to the occupational titles by the last known occupation prior to retirement: upper-grade nonmanual workers (e.g., teachers, physicians), lower-grade nonmanual workers (e.g., registered nurses, technicians), and manual workers (e.g., cleaners, maintenance workers). Additional background variables were: current smoking status (no vs yes), alcohol risk use (no vs yes [>24 units for men and >16 units for women per week]), physical activity (low vs high [≥14 metabolic equivalent hours per week), sleep difficulties (Jenkins Sleep Problem Scale, no vs yes [five to seven nights per week), work status (full-time vs part-time work/retirement), informal care to parent(s), spouse or adult child (yes/no), and number of chronic diseases (no diseases, one disease, or more than one disease) diagnosed by a physician (angina pectoris, myocardial infarction, cerebrovascular disease, osteoarthritis, rheumatoid arthritis, diabetes, asthma, chronic bronchitis and cancer).

### Statistical Analyses

Changes in psychological distress around retirement were assessed using negative binomial regression analyses, due to nonnormality of distribution and overdispersion, with generalized estimating equations (GEEs). As repeated measurements were used, the GEE model controlled for the intraindividual correlation between repeated measurements. The model uses an exchangeable correlation structure and is not sensitive to measurements missing completely at random. We conducted multiplicative and additive analyses with log-link and identity-link, respectively, to analyze relative and absolute changes in psychological distress during retirement transition. The absolute values were calculated, because we were interested in the magnitude of the change during retirement in addition to the relative changes taking into account preretirement psychological distress levels.

To analyze the changes in psychological distress during the retirement transition in relation to different contexts, we conducted separate analyses for each work-related/social/cumulative risk variable and included an interaction term between the variable and time (wave −1 [preretirement] and wave +1 [postretirement]). The adjusted rate ratios (multiplicative test) and rate differences (additive test) and their 95% confidence limits (CLs) were calculated by using contrast statements to represent an average of a 1-year change in psychological distress. The analyses were adjusted for age, gender, and occupational status at the study wave −1. Finally, additional analyses examined whether the associations during the retirement transition were confounded by lifestyle factors, work status, informal care, and number of chronic diseases. As the majority of study participants were women, all analyses originally included gender interactions. However, the results were similar among both gender (all interactions *p* >.05), and therefore men and women were combined in the analyses.

All analyses were conducted using the SAS 9.4 Statistical Package (SAS Institute Inc., Cary, North Carolina).

## Results

Characteristics of the study population prior to retirement, at the study wave −1 (between 2013 and 2018), are shown in Table 1. The mean age was 63.34 (*SD* 1.37) (range 58–68), and the majority of the study population were women (83%), working full-time (72%), and married (71%). The mean level of psychological distress was low (1.27 [*SD* 2.40]) and only 10% of the population reported psychological distress values ≥4 (a proxy for common mental disorders). Younger respondents, women, and nonmanual workers had higher psychological distress. Psychosocial work-related indicators, social living environment, health-related behaviors, and chronic diseases were also associated with psychological distress prior to retirement (Table 1).

**Table 1.** Characteristics of the Study Population Before Retirement and the Rate Ratios and Their 95% Confidence Limits (CLs) on Incidence of Psychological Distress

Characteristics	*N*	%/mean (*SD*)	Rate ratio of psychological distress (95% CL)
Psychological distress	3,338	1.27 (2.40)	
Gender			
Men	563	16.9	1.00
Women	2,775	83.1	1.18 (0.98, 1.43)
Age	3,338	63.34 (1.37)	0.92 (0.87, 0.97)
Occupational status			
Upper grade nonmanual	1,117	33.5	1.00
Lower grade nonmanual	1,027	30.8	1.07 (0.90, 1.28)
Manual	1,194	35.8	0.88 (0.74, 1.05)
Job demands			
Low	653	21.0	1.00
Middle	1,780	57.4	1.26 (1.05, 1.53)
High	671	21.6	1.77 (1.42, 2.22)
Skill discretion			
Low	633	20.5	1.62 (1.27, 2.07)
Middle	1,848	59.8	1.25 (1.02, 1.53)
High	610	19.7	1.00
Decision authority			
Low	657	21.2	2.52 (2.00, 3.17)
Middle	1,726	55.6	1.74 (1.44, 2.11)
High	721	23.2	1.00
Job strain			
No	2,480	80.1	1.00
Yes	618	20.0	1.83 (1.53, 2.20)
Neighborhood socioeconomic disadvantage			
Low	1,208	38.8	1.00
Middle	1,423	45.7	1.06 (0.91, 1.24)
High	486	15.6	1.20 (0.97, 1.50)
Neighborhood social cohesion			
Low	377	11.4	2.38 (1.90, 2.98)
Middle	1,494	45.0	1.46 (1.25, 1.69)
High	1,448	43.6	1.00
Marital status			
Married or cohabiting	2,325	71.4	1.00
Living alone	930	28.6	1.33 (1.13, 1.55)
Social network size			
Small	459	13.8	1.94 (1.57, 2.39)
Middle	1,322	39.8	1.27 (1.09, 1.48)
Large	1,542	46.4	1.00
Cumulative risks, psychosocial working conditions			
0	1,570	50.6	1.00
1	836	26.9	1.45 (1.22, 1.72)
≥2	698	22.5	2.06 (1.71, 2.48)
Cumulative risks, social living environment			
0	1,682	50.4	1.00
1	1,161	34.8	1.22 (1.05, 1.42)
≥2	495	14.8	2.07 (1.69, 2.53)
Cumulative risks, psychosocial working conditions, and social living environment			
0	959	29.0	1.00
1	1,021	30.9	1.14 (0.94, 1.37)
2	646	19.5	1.57 (1.28, 1.93)
3	374	11.3	2.05 (1.61, 2.61)
≥4	307	9.3	2.89 (2.23, 3.74)
Alcohol risk use			
No	3,052	91.9	1.00
Yes	270	8.1	1.17 (0.90, 1.51)
Smoking			
No	2,983	91.2	1.00
Yes	289	8.8	1.38 (1.08, 1.76)
Physical activity			
Low	1,247	37.7	1.42 (1.23, 1.64)
High	2,065	62.4	1.00
Sleep difficulties			
No	2,398	72.0	1.00
Yes	931	28.0	2.27 (1.95, 2.63)
Work status			
Full-time	2,387	71.5	1.00
Part-time work and/or retirement	951	28.5	1.21 (1.03, 1.41)
Chronic diseases			
0	1,096	34.1	1.00
1	1,369	42.6	1.37 (1.16, 1.61)
>1	747	23.3	1.71 (1.41, 2.07)
Informal care			
No	2,785	85.0	1.00
Yes	491	15.0	1.15 (0.94, 1.40)

*Note*: Analyses were adjusted for gender, age, and occupational status prior to retirement.

### Psychosocial Working Conditions

**Figure 1.**
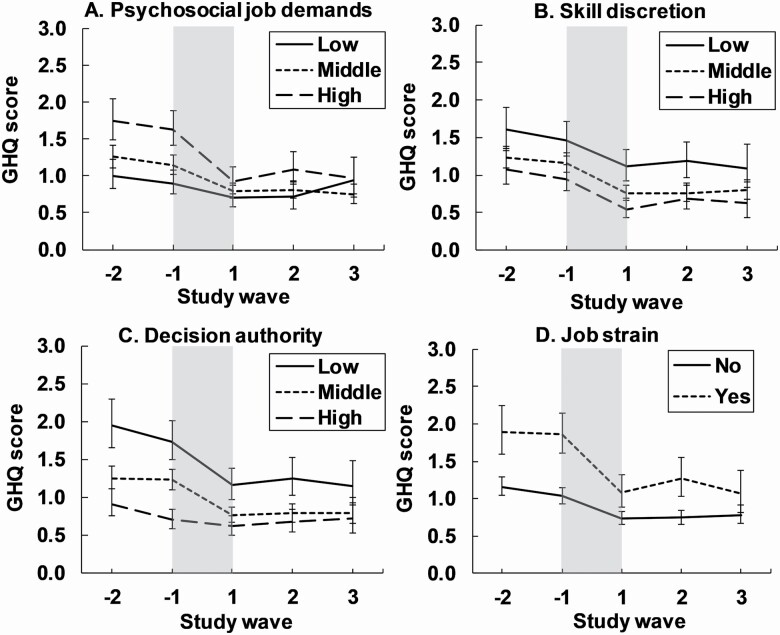
Mean psychological distress (12-item General Health Questionnaire [GHQ-12] score; 95% confidence intervals) in relation to retirement and psychosocial working conditions (A−D). The period of the retirement transition is highlighted in gray and is about 1 year. Models were adjusted for gender, age and occupational status prior to retirement.

In general, psychological distress decreased during the retirement transition, but greater relative and absolute decreases were observed for high job demands compared to low job demands (test for interaction: Job demands × Time: *p* = .016 for relative change and *p* < .001 for absolute change; Table 2 [A]; Figure 1A). This resulted in smaller postretirement differences in psychological distress for participants with high compared to low demands (differences at the study wave −1 vs +1: 0.78 [95% CL: 0.52, 1.04] vs 0.26 [95% CL: 0.05, 0.48]). The changes in psychological distress during the retirement transition did not depend on preretirement skill discretion (tests for interactions: *p* > .05 for relative and absolute change; Table 2 [A]; Figure 1B). Low decision authority was associated with greater relative and absolute decreases in psychological distress during the retirement transition compared to high decision authority (test for interaction: *p* = .026 for relative change and *p* < .001 for absolute change; Table 2 [A]; Figure 1C). Therefore, the differences between the low and high decision authority diminished after the retirement transition (differences between groups at the study wave −1 vs +1: 1.04 [95% CL: 0.78, 1.30] vs 0.54 [95% CL: 0.30, 0.77]). Finally, job strain was associated with greater relative and absolute changes in psychological distress during the retirement transition (interaction *p* = .025 for relative change and *p* < .001 for absolute change; Table 2 [A]; Figure 1D). The unequal decrease in psychological distress reduced the differences between the job strain categories after retirement (differences between the groups at the study wave −1 vs +1: 0.84 [95% CL: 0.59, 1.08] vs 0.34 [95% CL: 0.13, 0.55]). The additional analyses including additional confounders (smoking status, alcohol risk use, physical activity, sleep difficulties, work status, informal care status, and chronic diseases) showed similar results (Supplementary Table 2A).

**Table 2.** Changes in Psychological Distress During the Retirement Transition Period by Preretirement Psychosocial Working Conditions (A), Social Living Environment (B), and Cumulative Risk Factors (C)

Term	Before retirement	Retirement transition		Before retirement	Retirement transition	
		Relative changes			Absolute changes	
	Average psychological distress (95% CL)	Interaction *p*	Rate ratio (95% CL)	Average psychological distress (95% CL)	Interaction *p*	Rate difference (95% CL)
A. Psychosocial working conditions						
* *Job demands, *N* = 3,104		.016			<.001	
Low	0.91 (0.76, 1.08)		0.78 (0.64, 0.95)	0.92 (0.76, 1.08)		−0.24 (−0.40, −0.08)
Middle	1.17 (1.04, 1.30)		0.68 (0.61, 0.77)	1.19 (1.07, 1.31)		−0.39 (−0.51, −0.28)
High	1.66 (1.44, 1.92)		0.56 (0.49, 0.65)	1.70 (1.48, 1.91)		−0.76 (−0.93, −0.58)
* *Skill discretion, *N* = 3,091		.12			.88	
Low	1.48 (1.26, 1.73)		0.76 (0.64, 0.89)	1.51 (1.29, 1.73)		−0.39 (−0.60, −0.18)
Middle	1.18 (1.06, 1.31)		0.65 (0.59, 0.72)	1.20 (1.08, 1.32)		−0.45 (−0.55, −0.35)
High	0.97 (0.81, 1.17)		0.57 (0.46, 0.71)	1.01 (0.83, 1.20)		−0.45 (−0.62, −0.28)
* *Decision authority, *N* = 3,104		.026			<.001	
Low	1.76 (1.52, 2.04)		0.67 (0.58, 0.76)	1.77 (1.54, 2.01)		−0.61 (−0.80, −0.41)
Middle	1.25 (1.12, 1.39)		0.62 (0.55, 0.69)	1.26 (1.14, 1.39)		−0.50 (−0.61, −0.39)
High	0.71 (0.60, 0.85)		0.87 (0.71, 1.07)	0.73 (0.60, 0.86)		−0.10 (−0.24, −0.04)
Job strain, *N* = 3,098		.025			<.001	
No	1.05 (0.95, 1.16)		0.71 (0.64, 0.78)	1.07 (0.97, 1.17)		−0.33 (−0.41, −0.24)
Yes	1.89 (1.63, 2.18)		0.58 (0.50, 0.67)	1.90 (1.67, 2.14)		−0.83 (−1.04, −0.62)
B. Social living environment						
Neighborhood socioeconomic disadvantage, *N* = 3,117		.95			.91	
Low	1.15 (1.02, 1.31)		0.66 (0.58, 0.76)	1.19 (1.05, 1.33)		−0.42 (−0.55, −0.29)
Middle	1.21 (1.08, 1.36)		0.66 (0.59, 0.75)	1.25 (1.12, 1.39)		−0.45 (−0.58, −0.33)
High	1.35 (1.13, 1.61)		0.69 (0.58, 0.81)	1.37 (1.14, 1.61)		−0.46 (−0.66, −0.27)
Neighborhood social cohesion, *N* = 3,319		.43			.007	
Low	2.11 (1.79, 2.47)		0.68 (0.57, 0.81)	2.17 (1.84, 2.49)		−0.72 (−1.04, −0.41)
Middle	1.29 (1.16, 1.45)		0.62 (0.56, 0,70)	1.31 (1.18, 1.44)		−0.51 (−0.63, −0.40)
High	0.91 (0.80, 1.03)		0.70 (0.61, 0.80)	0.93 (0.82, 1.05)		−0.31 (−0.42, −0.20)
* *Marital status, *N* = 3,255		.49			.48	
Married or cohabiting	1.13 (1.03, 1.25)		0.64 (0.58, 0.71)	1.17 (1.06, 1.27)		−0.43 (−0.53, −0.34)
Living alone	1.46 (1.27, 1.67)		0.68 (0.60, 0.77)	1.49 (1.30, 1.68)		−0.50 (−0.66, −0.34)
Social network size, *N* = 3,323		.17			.031	
Small	1.86 (1.60, 2.17)		0.61 (0.51, 0.72)	1.91 (1.63, 2.20)		−0.75 (−1.00, −0.51)
Middle	1.23 (1.09, 1.39)		0.72 (0.64, 0.81)	1.27 (1.13, 1.41)		−0.39 (−0.51, −0.26)
Large	0.99 (0.88, 1.12)		0.62 (0.54, 0.71)	1.05 (0.93, 1.17)		−0.42 (−0.53, −0.31)
C. Cumulative risk factors						
* *Psychosocial working conditions, *N* = 3,104		.41			<.001	
0	0.91 (0.80, 1.03)		0.72 (0.63, 0.81)	0.91 (0.80, 1.02)		−0.28 (−0.38, −0.18)
1	1.32 (1.15, 1.52)		0.63 (0.54, 0.75)	1.35 (1.17, 1.53)		−0.49 (−0.67, −0.31)
≥2	1.83 (1.59, 2.11)		0.64 (0.56, 0.74)	1.82 (1.60, 2.05)		−0.67 (−0.86, −0.48)
Social living environment, *N* = 3,338		.71			.048	
0	1.01 (0.90, 1.13)		0.64 (0.56, 0.72)	1.05 (0.94, 1.17)		−0.40 (−0.50, −0.29)
1	1.22 (1.07, 1.38)		0.69 (0.60, 0.78)	1.25 (1.10, 1.40)		−0.41 (−0.54, −0.28)
≥2	2.01 (1.73, 2.33)		0.65 (0.56, 0.76)	2.05 (1.77, 2.33)		−0.73 (−0.97, −0.48)
Psychosocial working conditions and social living environment, *N* = 3,307		.57			<.001	
0	0.89 (0.77, 1.03)		0.64 (0.53, 0.76)	0.91 (0.77, 1.04)		−0.34 (−0.47, −0.22)
1	0.99 (0.86, 1.14)		0.75 (0.64, 0.87)	1.01 (0.88, 1.15)		−0.25 (−0.38, −0.12)
2	1.38 (1.19, 1.59)		0.64 (0.54, 0.76)	1.39 (1.20, 1.59)		−0.52 (−0.71, −0.33)
3	1.78 (1.48, 2.14)		0.64 (0.53, 0.78)	1.79 (1.49, 2.09)		−0.65 (−0.92, −0.37)
≥4	2.44 (2.05, 2.90)		0.63 (0.53, 0.75)	2.40 (2.03, 2.77)		−0.90 (−1.22, −0.59)

*Notes*: The adjusted rate ratios (multiplicative test) and rate differences (additive test) and their 95% confidence limits (CLs) are shown to represent an average of a 1-year change in psychological distress. Analyses were adjusted for gender, age, and occupational status prior to retirement.

### Social Living Environment

**Figure 2.**
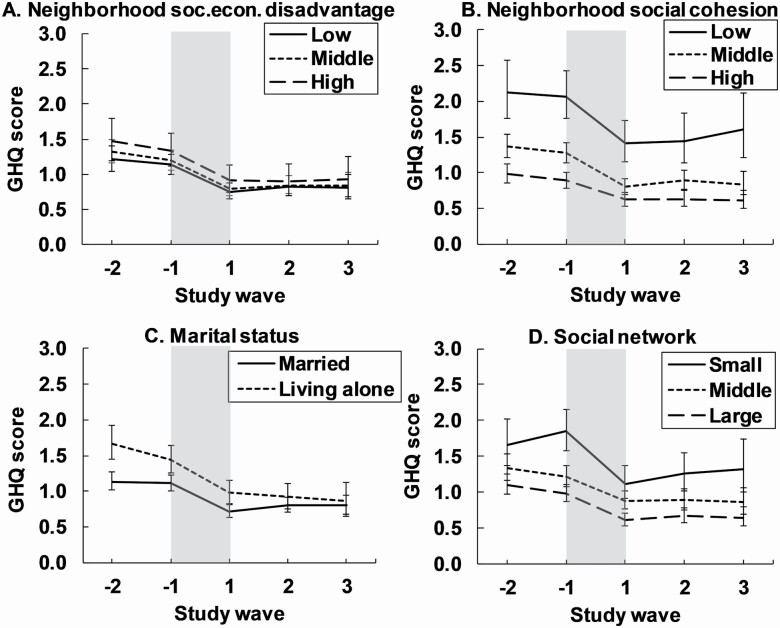
Mean psychological distress (12-item General Health Questionnaire [GHQ-12] score; 95% confidence intervals) in relation to retirement and social living environment (A−D). The period of the retirement transition is highlighted in gray and is about 1 year. Models were adjusted for gender, age and occupational status prior to retirement.

The changes in psychological distress during the retirement transition did not depend on neighborhood socioeconomic disadvantage (tests for interactions: *p* > .05 for relative and absolute changes; Table 2 [B]; Figure 2A). Low neighborhood social cohesion was associated with greater absolute changes (but not relative changes; interactions *p* = .007, *p* = .43, respectively) in psychological distress during the retirement transition compared to high neighborhood social cohesion (Table 2 [B]; Figure 2B). As a result, the gap between the categories in psychological distress after retirement was only slightly diminished (differences at the study wave −1 vs +1: 1.23 [95% CL: 0.90, 1.57] vs 0.82 [95% CL: 0.53, 1.12]). The changes in psychological distress during the retirement transition were largely independent on marital status (tests for interactions: *p* > .05 for relative and absolute change; Table 2 [B]; Figure 2C). Small social networks were associated with greater absolute changes (but not relative changes) in psychological distress during the retirement transition compared to larger social networks (interactions *p* = .031, *p* = .17, respectively; Table 2 [B]; Figure 2D). However, the psychological distress was still clearly higher after the retirement transition (study wave +1) in the small compared to large social network (differences between the groups at the study wave −1 vs +1: 0.87 [95% CL: 0.57, 1.17] vs 0.53 [95% CL: 0.29, 0.78]). The additional analyses adjusting for additional confounders showed similar results (Supplementary Table 2B).

### Cumulative Risk Factors

**Figure 3.**
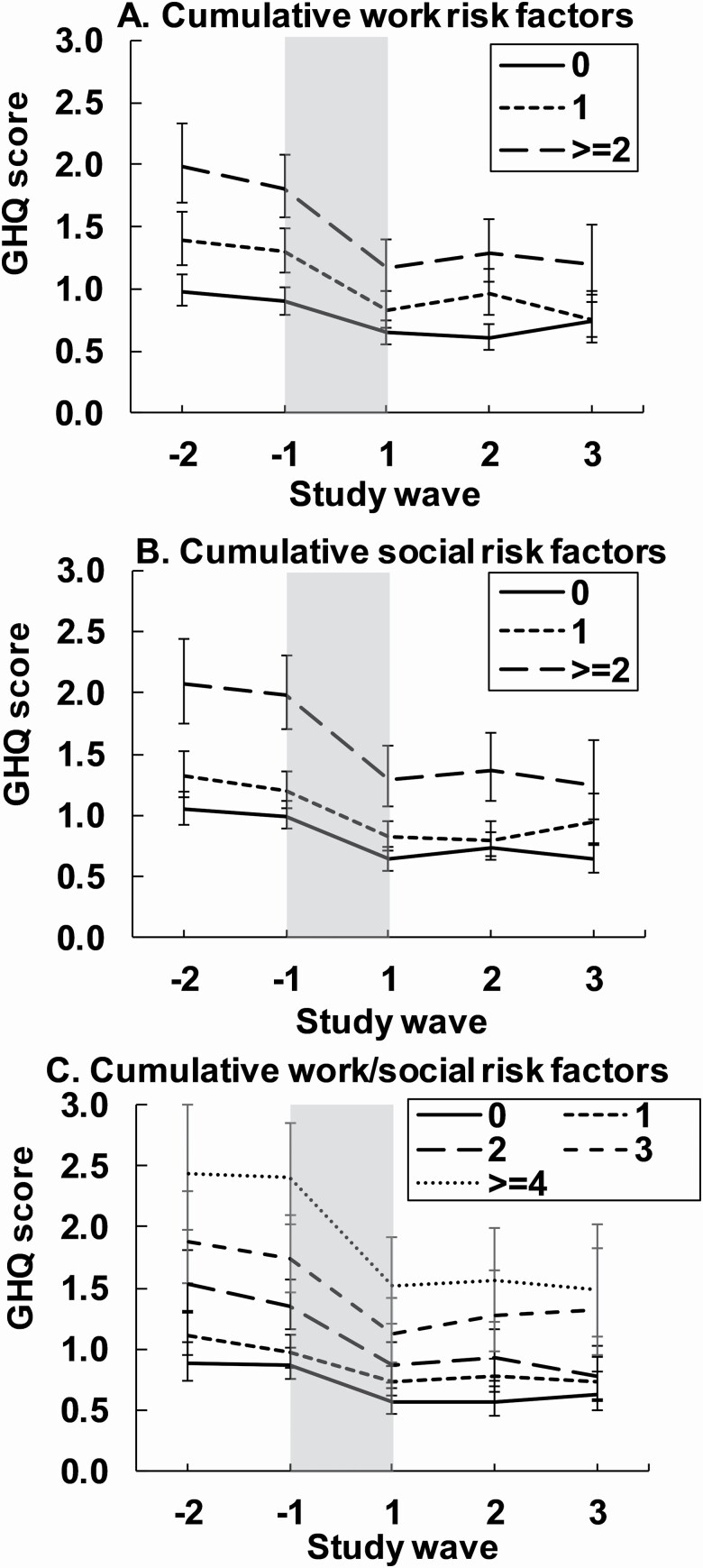
Mean psychological distress (12-item General Health Questionnaire [GHQ-12] score; 95% confidence intervals) in relation to retirement and cumulative risk factors in psychosocial working conditions (A), social living environment (B) and both (C). The period of the retirement transition is highlighted in gray and is about 1 year. Models were adjusted for gender, age and occupational status prior to retirement.

Having two or more job-related risk factors (high job demands/low skill discretion/low decision authority/job strain) led to a greater absolute decrease (but not relative change) in psychological distress during the retirement transition compared to having no job-related risk factors (Table 2 [C]; Figure 3A; interactions absolute change *p* < .001, relative change *p* = .41). Similarly, having two or more social risk factors (high neighborhood socioeconomic disadvantage/low social cohesion/single/small social network size) resulted in a greater absolute change (but not relative change) in psychological distress during the retirement transition compared to having no or only one social risk factor (interactions absolute change: *p* = .048, relative change *p* = .71; Table 2 [C]; Figure 3B). Finally, having four or more job-related and/or social living environment risk factors lead to greater absolute change (but not relative change) in psychological distress than having fewer or no risk factors (interactions absolute change *p* < .001, relative change *p* = .57; Table 2 [C]; Figure 3C). As a result, the unequal decreases during the retirement transition led to those having more risks still reporting higher psychological distress postretirement than those with no risks and the differences diminished only slightly (differences at the study wave −1 vs +1: work [two or more risks vs no risks] 0.91 [95% CL: 0.68, 1.15] vs 0.52 [95% CL: 0.32, 0.73]; social [two or more risks vs no risks]: 1.00 [95% CL: 0.70, 1.30] vs 0.67 [95% CL: 0.42, 0.92]; work/social [four or more risks vs no risks]: 1.49 [95% CL: 1.11, 1.88] vs 0.93 [95% CL: 0.60, 1.26]). The additional analyses showed similar results (Supplementary Table 2C).

## Discussion

This longitudinal study among Finnish public sector employees shows that changes in psychological distress during the retirement transition depend on the contexts the individuals retire from, which is in line with the life-course perspective (Wang, 2007) and social buffering theory (Cohen, 2004). To our knowledge, this is the first study investigating the specific effects of neighborhood and individual-level characteristics of social living environment on psychological distress following the retirement transition. As expected, retirement was associated with decreases in psychological distress in line with previous studies (Jokela et al., 2010; Oshio & Kan, 2017; Van Der Heide et al., 2013; Westerlund et al., 2010).

A novel finding of our study was that the improvements occur very soon after the retirement transition. This may reflect the importance of the genericity of the retirement systems, improved health behavior (Ding et al., 2016; Myllyntausta et al., 2017; Oshio & Kan, 2017; Stenholm et al., 2016; Vahtera et al., 2009), and relief from work-related stressors following retirement. However, the improvements in psychological distress following retirement distributed unequally to the participants and depended on the psychosocial working conditions, social living environment, as well as accumulation of risks prior to retirement.

### Psychosocial Working Conditions

We found support for our first hypothesis and recent study findings among British civil servants (Fleischmann et al., 2020), suggesting that people retiring from poorer psychosocial working conditions benefit most regarding their mental health during the retirement transition. We found that high psychosocial job demands, low decision authority, and job strain predicted higher decrease in psychological distress during the retirement transition. This is not only due to participants with poor psychosocial working conditions reporting higher levels of psychological distress prior to retirement, as both the absolute and relative changes during the retirement transition were higher in participants coming from poorer working conditions. Skill discretion was the only work-related factor that was not associated with the changes in psychological distress during the retirement transition, further supporting the recent findings (Fleischmann et al., 2020). The differences in postretirement psychological distress were smaller in participants from poorer compared to better working conditions than before retirement, suggesting that people recover relatively soon from work-related stressors after retirement. Similar association have been observed in self-rated health with greater retirement-related improvement for those retiring from poor work environment (Van Den Bogaard et al., 2016; Westerlund et al., 2009), further underlining the importance of psychosocial working conditions on postretirement health.

### Social Living Environment

Social living environment has received much less attention in relation to the retirement transition and mental health despite the wide literature on substantial effects of social determinants on mental health in older age (Vyas & Okereke, 2020) or on timing of retirement (e.g., Wang & Shi, 2014). Contrarily to work-related stressors, which relieve immediately after retirement, social living environment stays relatively the same after retirement. Only small decreases in social network sizes have been observed during the retirement transition (Fletcher, 2014), potentially as a part of normal decrease in social network sizes with age (Wrzus et al., 2013). Furthermore, as our main analyses were conducted within 1 year after retirement, major changes in residential neighborhoods were unlikely. Thus, the social stressors remain constant throughout the retirement transition, explaining the persistent level differences between those retirees with more and fewer social relationships.

These findings are in line with the extensive literature showing that the lack of social relationships predicts poor mental health. However, the results do not support social buffering hypothesis according to which social relationships buffer the effects of stressful life events and are beneficial for those suffering adversity, but do not play a role in health for those without highly stressful demands (e.g., Cohen, 2004). Because people experienced overall decreases in psychological distress during the retirement transition, at least on a population level, retirement can be viewed as a positive rather than stressful life event, in line with the previous studies in the same population (e.g., Stenholm & Vahtera, 2017). This may explain why those with more social relationships did not benefit more during the retirement transition than those with fewer social relationships. The results, thus, contradict our second hypothesis. On the other hand, there were some signs of those from poorer social contexts (low neighborhood social cohesion and small social networks) benefitting more from the retirement, in line with previous studies (Schuring et al., 2015; Westerlund et al., 2009). However, as the relative changes between the groups with more and fewer social relationships were not different, the observed differences were likely due to higher levels already before retirement. This suggests that social relationships have a general positive effects on mental health (main effect), irrespective of whether one is under stress (Cohen, 2004). Moreover, people with fewer social relationships have been found to report higher job-related stress (Shin & Lee, 2016), and thus the greater reductions in psychological distress may also reflect relief from greater burden of work-related stressors. Nevertheless, substantial differences in psychological distress still exist after the retirement transition for the benefit of those with more social relationships.

The results gained from social cohesion (as a neighborhood-level measure) and social network size (as an individual-level measure) show that measuring social living environment in multiple levels is important. However, marital status was not associated with the changes in psychological distress during the retirement transition. Married (or cohabiting) and single individuals benefit equally from the retirement, in line with previous studies (Kolodziej & García-Gómez, 2019; Westerlund et al., 2010). However, a more complex association between marital status, retirement, and mental health may still exist, as a recent change in relationship status (divorce/widowhood) is related to late-life depression risk (Vyas & Okereke, 2020). Psychological distress levels were neither associated with neighborhood socioeconomic disadvantage prior to retirement nor during the retirement transition. This may be related to low average psychological distress levels in the study population, while studies more representative of the general population have observed that the presence of mental disorders has been associated with lower neighborhood socioeconomic status also in Finland (Kivimäki et al., 2020).

### Cumulative Risk Factors

Most research on mental health risk factors has usually focused on single exposures, although risk factors are found in multiple contexts and tend to cluster across the life course or at a certain life stage (Alegría et al., 2018). We found partial support for our third hypothesis by observing that when exposed to several adverse contexts simultaneously, measured as work-related risk factors or social risk factors or as both, psychological distress was higher, but also the decreases were greater during the retirement transition compared to fewer or no risk factors. However, prominent differences still remain after the retirement transition, and those with more risks continue to have higher psychological distress. Interestingly, this concerns also work-related risk factors, although the participants are no longer exposed to them after retirement. This suggests that when exposed to several stressors at the same time from different contexts, the negative effects on mental health are long term, and even the relief from these stressors will not lead to equally low psychological distress than without these stressors. Further, the results show that accumulation of several risk factors from social and working environment at the same time is particularly harmful for mental health, indicated by the highest levels in psychological distress throughout the retirement period.

### Strengths and Limitations

The strength of this study was the use of very recent and large longitudinal data with repeated measurements on psychological distress before and after retirement, and multiple psychosocial working condition and social environment measures, which enabled us to investigate how different contexts are associated with changes in mental health during the retirement transition. Most of the studies to date investigating changes in mental health during the retirement transition have used cross-sectional data comparing those retiring to those continuing at work (e.g., Vyas and Okereke, 2020) or concentrated on general patterns and not taking into account the contexts people are retiring from (but see Åhlin et al., 2020; Fleischmann et al., 2020; Wang, 2007). Because all participants retired based on statutory age-based retirement, ill-health leading to retirement decision was an unlikely source of bias. Moreover, although the study population was female-dominated, there were no interactions between risk factors and gender, and the cohort is a good representation of the potential variability in the occupations in municipal sector in Finland. However, further studies are needed in other study populations and countries (also controlling for individual income levels, which we were unable to include in the analyses), as the beneficial effect of the retirement transition on mental health and the association with different contexts may be limited to relatively favorable pension systems of Scandinavian welfare countries (Richardson et al., 2019). Also, as the psychological distress levels in the study population were relatively low, more studies among people with more severe mental health problems are needed. Furthermore, we cannot put aside reverse causality. People with compromised mental health are more likely to view their surroundings more negatively and may self-report their working conditions poorer than they actually are (Kivimäki et al., 2010). However, this is unlikely to explain the finding that people retiring from poorer psychosocial working conditions benefit most from the retirement transition as individual differences in reporting are likely to be relatively stable over time. The same concerns social living environment as one’s mental health is known to affect the formation and maintenance of social interactions and social relationships (Schaefer et al., 2011). The observed associations may thus be bidirectional and operate concurrently, resulting in reinforcing feedback loops between poor mental health and lack of social relationships (Jin et al., 2020).

## Conclusions

Psychological distress decreased during the retirement transition, but the magnitude of the change was dependent on psychosocial working environment and social living environment, and the participants retiring from poorer contexts benefit more than the participants retiring from better contexts. Furthermore, the results suggest that poor psychosocial working conditions have shorter-term effect than poor social and cumulative living environment, which have longer-term prevailing effects on mental health. More studies incorporating life-long cumulative aspect and composite effect of exposures across multiple levels (e.g., individual and neighborhood) and contexts (physical, social, and work-related) are urged to clarify the causal pathways, those at risk, and the most important associations determining mental health during the retirement transition and more generally at older ages.

## Funding

This work was supported by the Academy of Finland (286294, 319246, and 294154 to S. Stenholm; 321409 and 329240 to J. Vahtera); Finnish Work Environment Fund (118060 to S. Stenholm; 190172 to M. Virtanen); and NordForsk (70521 to J. Vahtera). The funders had no role in study design, data collection and analysis, decision to publish, or preparation of the manuscript.

## Conflict of Interest

None declared.

## Supplementary Material

gbab054_suppl_Supplementary_Material

## References

[CIT0001] Åhlin, J. K., Peristera, P., Westerlund, H., & Magnusson Hanson, L. L. (2020). Psychosocial working characteristics before retirement and depressive symptoms across the retirement transition: A longitudinal latent class analysis. Scandinavian Journal of Work, Environment & Health, 46(5), 488–497. doi:10.5271/sjweh.3889 PMC7737805 32091111

[CIT0002] Alegría, M., NeMoyer, A., Falgàs Bagué, I., Wang, Y., & Alvarez, K. (2018). Social determinants of mental health: Where we are and where we need to go. Current Psychiatry Reports, 20(11), 95. doi:10.1007/s11920-018-0969-9 30221308 PMC6181118

[CIT0003] Antonucci, T. C . (1986). Measuring social support networks: Hierarchial mapping technique. Generations: Journal of the American Society of Aging, 10(4), 10–12. Retrieved April 21, 2021, from http://www.jstor.org/stable/44876253

[CIT0004] Baranyi, G., Sieber, S., Cullati, S., Pearce, J. R., Dibben, C. J. L., & Courvoisier, D. S. (2020). The longitudinal associations of perceived neighborhood disorder and lack of social cohesion with depression among adults aged 50 years or older: An individual-participant-data meta-analysis from 16 high-income countries. American Journal of Epidemiology, 189(4), 343–353. doi:10.1093/aje/kwz209 31573028 PMC7274178

[CIT0005] Bulloch, A. G. M., Williams, J. V. A., Lavorato, D. H., & Patten, S. B. (2017). The depression and marital status relationship is modified by both age and gender. Journal of Affective Disorders, 223, 65–68. doi:10.1016/j.jad.2017.06.007 28732242

[CIT0006] Cohen, S . (2004). Social relationships and health. The American Psychologist, 59(8), 676–684. doi:10.1037/0003-066X.59.8.676 15554821

[CIT0007] Dave, D., Rashad, I., & Spasojevic, J. (2008). The effects of retirement on physical and mental health outcomes. Southern Economic Journal, 75(2), 497–523. doi:10.3386/w12123

[CIT0008] Degnan, A., Berry, K., Sweet, D., Abel, K., Crossley, N., & Edge, D. (2018). Social networks and symptomatic and functional outcomes in schizophrenia: A systematic review and meta-analysis. Social Psychiatry and Psychiatric Epidemiology, 53(9), 873–888. doi:10.1007/s00127-018-1552-8 29951929 PMC6133157

[CIT0009] De Silva, M. J., McKenzie, K., Harpham, T., & Huttly, S. R. (2005). Social capital and mental illness: A systematic review. Journal of Epidemiology and Community Health, 59(8), 619–627. doi:10.1136/jech.2004.029678 16020636 PMC1733100

[CIT0010] Ding, D., Grunseit, A. C., Chau, J. Y., Vo, K., Byles, J., & Bauman, A. E. (2016). Retirement—A transition to a healthier lifestyle? Evidence from a large Australian study. American Journal of Preventive Medicine, 51(2), 170–178. doi:10.1016/j.amepre.2016.01.019 26972491

[CIT0011] Evans, G. W . (2003). The built environment and mental health. Journal of Urban Health: Bulletin of the New York Academy of Medicine, 80(4), 536–555. doi:10.1093/jurban/jtg063 14709704 PMC3456225

[CIT0012] Fleischmann, M., Xue, B., & Head, J. (2020). Mental health before and after retirement—Assessing the relevance of psychosocial working conditions: The Whitehall II prospective study of British civil servants. The Journals of Gerontology, Series B: Psychological Sciences and Social Sciences, 75(2), 403–413. doi:10.1093/geronb/gbz042 PMC7392102 31100154

[CIT0013] Fletcher, J. M . (2014). Late life transitions and social networks: The case of retirement. Economics Letters, 125(3), 459–462. doi:10.1016/j.econlet.2014.10.004

[CIT0014] Goldberg, D . (1972). The detection of psychiatric illness by questionnaire: A technique for the identification and assessment of non-psychotic psychiatric illness. Oxford University Press.

[CIT0015] Habibi, E., Poorabdian, S., & Shakerian, M. (2015). Job strain (demands and control model) as a predictor of cardiovascular risk factors among petrochemical personnel. Journal of Education and Health Promotion, 4(1), 16. doi:10.4103/2277-9531.154034 PMC4389361 25861661

[CIT0016] Harvey, S. B., Sellahewa, D. A., Wang, M. J., Milligan-Saville, J., Bryan, B. T., Henderson, M., Hatch, S. L., & Mykletun, A. (2018). The role of job strain in understanding midlife common mental disorder: A national birth cohort study. The Lancet. Psychiatry, 5(6), 498–506. doi:10.1016/S2215-0366(18)30137-8 29754990

[CIT0017] Jin, Y., Zhu, D., & He, P. (2020). Social causation or social selection? The longitudinal interrelationship between poverty and depressive symptoms in China. Social Science & Medicine (1982), 249, 112848. doi:10.1016/j.socscimed.2020.112848 32087488

[CIT0018] Joensuu, M., Kivimäki, M., Pentti, J., Virtanen, M., Väänänen, A., & Vahtera, J. (2014). Components of job control and mortality: The Finnish Public Sector Study. Occupational and Environmental Medicine, 71(8), 536–542. doi:10.1136/oemed-2014-102111 24891558

[CIT0019] Jokela, M., Ferrie, J. E., Gimeno, D., Chandola, T., Shipley, M. J., Head, J., Vahtera, J., Westerlund, H., Marmot, M. G., & Kivimäki, M. (2010). From midlife to early old age: Health trajectories associated with retirement. Epidemiology (Cambridge, Mass.), 21(3), 284–290. doi:10.1097/EDE.0b013e3181d61f53 PMC3204317 20220519

[CIT0020] Kail, B. L., & Carr, D. C. (2020). Structural social support and changes in depression during the retirement transition: “I get by with a little help from my friends.” The Journals of Gerontology, Series B: Psychological Sciences and Social Sciences, 75(9), 2040–2049. doi:10.1093/geronb/gbz126 31606741

[CIT0021] Karasek, R., Brisson, C., Kawakami, N., Houtman, I., Bongers, P., & Amick, B. (1998). The Job Content Questionnaire (JCQ): An instrument for internationally comparative assessments of psychosocial job characteristics. Journal of Occupational Health Psychology, 3(4), 322–355. doi:10.1037//1076-8998.3.4.322 9805280

[CIT0022] Kauppi, M., Kawachi, I., Batty, G. D., Oksanen, T., Elovainio, M., Pentti, J., Aalto, V., Virtanen, M., Koskenvuo, M., Vahtera, J., & Kivimäki, M. (2018). Characteristics of social networks and mortality risk: Evidence from 2 prospective cohort studies. American Journal of Epidemiology, 187(4), 746–753. doi:10.1093/aje/kwx301 29020140

[CIT0023] Kivimäki, M., Batty, G. D., Pentti, J., Shipley, M. J., Sipilä, P. N., Nyberg, S. T., Suominen, S. B., Oksanen, T., Stenholm, S., Virtanen, M., Marmot, M. G., Singh-Manoux, A., Brunner, E. J., Lindbohm, J. V., Ferrie, J. E., & Vahtera, J. (2020). Association between socioeconomic status and the development of mental and physical health conditions in adulthood: A multi-cohort study. The Lancet. Public Health, 5(3), e140–e149. doi:10.1016/S2468-2667(19)30248-8 32007134

[CIT0024] Kivimäki, M., Hotopf, M., & Henderson, M. (2010). Do stressful working conditions cause psychiatric disorders? Occupational Medicine (Oxford, England), 60(2), 86–87. doi:10.1093/occmed/kqp183 20157188

[CIT0025] Kolodziej, I. W. K., & García-Gómez, P. (2019). Saved by retirement: Beyond the mean effect on mental health. Social Science & Medicine (1982), 225, 85–97. doi:10.1016/j.socscimed.2019.02.003 30822608

[CIT0026] Lawrence, D., Kisely, S., & Pais, J. (2010). The epidemiology of excess mortality in people with mental illness. Canadian Journal of Psychiatry, 55(12), 752–760. doi:10.1177/070674371005501202 21172095

[CIT0027] Leskinen, T., Pulakka, A., Heinonen, O. J., Pentti, J., Kivimäki, M., Vahtera, J., & Stenholm, S. (2018). Changes in non-occupational sedentary behaviours across the retirement transition: The Finnish Retirement and Aging (FIREA) study. Journal of Epidemiology and Community Health, 72(8), 695–701. doi:10.1136/jech-2017-209958 29636399

[CIT0028] Lund, C., Brooke-Sumner, C., Baingana, F., Baron, E. C., Breuer, E., Chandra, P., Haushofer, J., Herrman, H., Jordans, M., Kieling, C., Medina-Mora, M. E., Morgan, E., Omigbodun, O., Tol, W., Patel, V., & Saxena, S. (2018). Social determinants of mental disorders and the sustainable development goals: A systematic review of reviews. The Lancet. Psychiatry, 5(4), 357–369. doi:10.1016/S2215-0366(18)30060-9 29580610

[CIT0029] Myllyntausta, S., Salo, P., Kronholm, E., Aalto, V., Kivimäi, M., Vahtera, J., & Stenholm, S. (2017). Changes in sleep duration during transition to statutory retirement: A longitudinal cohort study. Sleep, 40(7), zsx087. doi:10.1093/sleep/zsx087 28541436

[CIT0030] Myllyntausta, S., Salo, P., Kronholm, E., Aalto, V., Pentti, J., Kivimäki, M., Vahtera, J., & Stenholm, S. (2018). Changes in sleep difficulties during the transition to statutory retirement. Sleep, 41(1), zsx182. doi:10.1093/sleep/zsx182 29155955

[CIT0031] Oshio, T., & Kan, M. (2017). The dynamic impact of retirement on health: Evidence from a nationwide ten-year panel survey in Japan. Preventive Medicine, 100, 287–293. doi:10.1016/j.ypmed.2017.04.007 28583660

[CIT0032] Richardson, S., Carr, E., Netuveli, G., & Sacker, A. (2019). Country-level welfare-state measures and change in wellbeing following work exit in early old age: Evidence from 16 European countries. International Journal of Epidemiology, 48(2), 389–401. doi:10.1093/ije/dyy205 30277529 PMC6469302

[CIT0033] Sampson, R. J., Raudenbush, S. W., & Earls, F. (1997). Neighborhoods and violent crime: A multilevel study of collective efficacy. Science (New York, N.Y.), 277(5328), 918–924. doi:10.1126/science.277.5328.918 9252316

[CIT0034] Schaefer, D. R., Kornienko, O., & Fox, A. M. (2011). Misery does not love company: Network selection mechanisms and depression homophily. American Sociological Review, 76(5), 764–785. doi:10.1177/0003122411420813

[CIT0035] Schuring, M., Robroek, S. J., Lingsma, H. F., & Burdorf, A. (2015). Educational differences in trajectories of self-rated health before, during, and after entering or leaving paid employment in the European workforce. Scandinavian Journal of Work, Environment & Health, 41(5), 441–450. doi:10.5271/sjweh.3514 26186611

[CIT0036] Scott, K. M., Lim, C., Al-Hamzawi, A., Alonso, J., Bruffaerts, R., Caldas-de-Almeida, J. M., Florescu, S., de Girolamo, G., Hu, C., de Jonge, P., Kawakami, N., Medina-Mora, M. E., Moskalewicz, J., Navarro-Mateu, F., O’Neill, S., Piazza, M., Posada-Villa, J., Torres, Y., & Kessler, R. C. (2016). Association of mental disorders with subsequent chronic physical conditions: World Mental Health Surveys from 17 countries. JAMA Psychiatry, 73(2), 150–158. doi:10.1001/jamapsychiatry.2015.2688 26719969 PMC5333921

[CIT0037] Shin, S. Y., & Lee, S. G. (2016). Effects of hospital workers’ friendship networks on job stress. PLoS ONE, 11(2), e0149428. doi:10.1371/journal.pone.0149428 26900945 PMC4763201

[CIT0038] Stenholm, S., Pulakka, A., Kawachi, I., Oksanen, T., Halonen, J. I., Aalto, V., Kivimäki, M., & Vahtera, J. (2016). Changes in physical activity during transition to retirement: A cohort study. The International Journal of Behavioral Nutrition and Physical Activity, 13, 51. doi:10.1186/s12966-016-0375-9 27084334 PMC4833915

[CIT0039] Stenholm, S., & Vahtera, J. (2017). Does retirement benefit health? Preventive Medicine, 100, 294–295. doi:10.1016/j.ypmed.2017.05.007 28583661

[CIT0040] Stenholm, S., Virtanen, M., Pentti, J., Oksanen, T., Kivimäki, M., & Vahtera, J. (2020). Trajectories of self-rated health before and after retirement: Evidence from two cohort studies. Occupational and Environmental Medicine, 77(2), 70–76. doi:10.1136/oemed-2019-106026 31826927

[CIT0041] Vahtera, J., Westerlund, H., Hall, M., Sjösten, N., Kivimäki, M., SalO, P., Ferrie, J. E., Jokela, M., Pentti, J., Singh-Manoux, A., Goldberg, M., & Zins, M. (2009). Effect of retirement on sleep disturbances: The GAZEL prospective cohort study. Sleep, 32(11), 1459–1466. doi:10.1093/sleep/32.11.1459 19928385 PMC2768952

[CIT0042] Van Den Bogaard, L., Henkens, K., & Kalmijn, M. (2016). Retirement as a relief? The role of physical job demands and psychological job stress for effects of retirement on self-rated health. European Sociological Review, 32(2), 295–306. doi:10.1093/esr/jcv135

[CIT0043] Van Der Heide, I., Van Rijn, R. M., Robroek, S. J., Burdorf, A., & Proper, K. I. (2013). Is retirement good for your health? A systematic review of longitudinal studies. BMC Public Health, 13, 1180. doi:10.1186/1471-2458-13-1180 24330730 PMC4029767

[CIT0044] Vyas, C. M., & Okereke, O. I. (2020). Late-life depression: A narrative review on risk factors and prevention. Harvard Review of Psychiatry, 28(2), 72–99. doi:10.1097/HRP.0000000000000240 31977599

[CIT0045] Wang, M . (2007). Profiling retirees in the retirement transition and adjustment process: Examining the longitudinal change patterns of retirees’ psychological well-being. Journal of Applied Psychology, 92(2), 455–474. doi:10.1037/0021-9010.92.2.455 17371091

[CIT0046] Wang, M., & Shi, J. (2014). Psychological research on retirement. Annual Review of Psychology, 65, 209–233. doi:10.1146/annurev-psych-010213-115131 23751036

[CIT0047] Westerlund, H., Kivimäki, M., Singh-Manoux, A., Melchior, M., Ferrie, J. E., Pentti, J., Jokela, M., Leineweber, C., Goldberg, M., Zins, M., & Vahtera, J. (2009). Self-rated health before and after retirement in France (GAZEL): A cohort study. Lancet (London, England), 374(9705), 1889–1896. doi:10.1016/S0140-6736(09)61570-1 19897238

[CIT0048] Westerlund, H., Vahtera, J., Ferrie, J. E., Singh-Manoux, A., Pentti, J., Melchior, M., Leineweber, C., Jokela, M., Siegrist, J., Goldberg, M., Zins, M., & Kivimäki, M. (2010). Effect of retirement on major chronic conditions and fatigue: French GAZEL occupational cohort study. BMJ (Clinical Research ed.), 341, c6149. doi:10.1136/bmj.c6149 PMC2990862 21098617

[CIT0049] Wheaton, B . (1990). Life transitions, role histories, and mental health. American Sociological Review, 55(2), 209–223. doi:10.2307/2095627

[CIT0050] Wrzus, C., Hänel, M., Wagner, J., & Neyer, F. J. (2013). Social network changes and life events across the life span: A meta-analysis. Psychological Bulletin, 139(1), 53–80. doi:10.1037/a0028601 22642230

