# Supplementary material for: Psychological Distress During the Retirement Transition and the Role of Psychosocial Working Conditions and Social Living Environment
Source: J Gerontol B Psychol Sci Soc Sci. 2021 Aug 16;77(1):135–48. doi: 10.1093/geronb/gbab054 (PMC8755891; doi:10.1093/geronb/gbab054)
Supplement: gbab054_suppl_Supplementary_Material [file gbab054_suppl_supplementary_material.docx]

SUPPLEMENTARY MATERIAL

**Psychological distress during the retirement transition and the role of psychosocial working conditions and social living environment**

**Supplementary Table 1.** Items of general health questionnaire (GHQ-12) and psychosocial working condition and social living environment measures.

| **GHQ-12 (4-point response scale (1 = not at all, 2 = same as usual, 3 = more than usual, and 4 = much more than usual))** |
| --- |
| Have you recently: |
| 1. Been able to concentrate on what you’re doing? |
| 2. Lost much sleep over worry? |
| 3. Felt you were playing a useful part in things? |
| 4. Felt capable of making decisions about things? |
| 5. Felt constantly under strain? |
| 6. Felt you couldn’t overcome your difficulties? |
| 7. Been able to enjoy your normal day-to-day activities? |
| 8. Been able to face up to your problems? |
| 9. Been feeling unhappy and depressed? |
| 10. Been losing confidence in yourself? |
| 11. Been thinking of yourself as a worthless person? |
| 12. Been feeling reasonably happy, all things considered |
| **A. Psychosocial working conditions**: |
| ***Job demands* (5-point response scale (strongly agree to strongly disagree))** |
| 1. The job requires working very fast |
| 2. The job requires working very hard |
| 3. The job involves an excessive amount of work |
| 4. The worker has enough time to get the job done |
| 5. The work is very hectic |
| ***Skill discretion* (5-point response scale (strongly agree to strongly disagree))** |
| 1. My job requires that I learn new things |
| 2. My job involves a lot of repetitive work |
| 3. My job requires me to be creative |
| 4. My job requires a high level of skill |
| 5. I get to do a variety of things on my job |
| 6. I have an opportunity to develop my own special abilities |
| **Decision authority (5-point response scale (strongly agree to strongly disagree))** |
| 1. My job allows me to make a lot of decisions on my own |
| 2. I have a lot to say about what happens in my job |
| 3. On my job I am given little freedom to decide how I do my work |
| **Job strain (combines job demands and job control)** |
| **Job control (5-point response scale (strongly agree to strongly disagree)). Combines skill discretion and decision authority.** |
| 1. My job allows me to make a lot of decisions on my own |
| 2. My job requires me to be creative |
| 3. My job requires that I learn new things |
| 4. My job involves a lot of repetitive work |
| 5. I have a lot of say about what happens on my job |
| 6. My job requires a high level of skill |
| 7. I get to do a variety of different things on my job |
| 8. I have an opportunity to develop my own special abilities |
| 9. On my job, I have very little freedom to decide how I do my work |
| **B. Social living environment:** |
| **Social cohesion (*5-point response scale (strongly agree to strongly disagree))*** |
| 1. People around here are willing to help their neighbors |
| 2. This is a close-knit neighborhood |
| 3. People in this neighborhood can be trusted |
| 4. People in this neigh­borhood generally do not get along with one another |
| 5. People in this neighborhood share the same values |
| **Social network (10-point response scale from 0 to over 20)** |
| 1. The number of people to whom you feel so close it is hard to imagine life without them |
| 2. The number of people who felt not quite that close but still important |
| 3. The number of people who had not already been mentioned and not that close but important enough to belong to the individual’s personal social network (regular interaction) |

**Supplementary Table 2.** Results of additional models showing changes in psychological distress during the retirement transition period by pre-retirement psychosocial working conditions (A), social living environment (B) and cumulative risk factors (C). The adjusted rate ratios (multiplicative test) and rate differences (additive test) and their 95% confidence intervals (CIs) are shown to represent an average of a 1-year change in psychological distress. Analyses were adjusted for gender, age, occupational status, life-style factors (smoking status, alcohol risk use, physical activity, sleep difficulties), work status, informal caregiver status and number of chronic diseases.

| Term | Before retirement | Retirement transition | | Before retirement | Retirement transition | |
| --- | --- | --- | --- | --- | --- | --- |
|  |  | Relative changes | |  | Absolute changes | |
|  | Average psych. distress [mean [95 % CL]] | Interaction p | Rate ratio [95% CL] | Average psych. distress [mean [95 % CL]] | Interaction p | Rate diff. [95% CL] |
| ***A. Psychosocial working conditions*** |  |  |  |  |  |  |
| *Job demands n=2865* |  | 0.045 |  |  | 0.0022 |  |
| Low | 1.37 [1.09, 1.73] |  | 0.78 [0.62, 0.96] | 1.40 [1.18, 1.62]^1)^ |  | -0.17 [-0.33, -0.01] |
| Middle | 1.73 [1.43, 2.10] |  | 0.67 [0.59, 0.76] | 1.60 [1.39, 1.80] |  | -0.34 [-0.44, -0.23] |
| High | 2.23 [1.81, 2.76] |  | 0.55 [0.46, 0.66] | 1.96 [1.70, 2.22] |  | -0.60 [-0.79, -0.42] |
| *Skill discretion n=2852* |  | 0.37 |  |  | 0.93 |  |
| Low | 2.04 [1.64, 2.53] |  | 0.73 [0.60, 0.88] | 1.81 [1.55, 2.06]^1)^ |  | -0.35 [-0.56, -0.14] |
| Middle | 1.68 [1.40, 2.03] |  | 0.64 [0.57, 0.73] | 1.58 [1.38, 1.77] |  | -0.36 [-0.46, -0.26] |
| High | 1.47 [1.15, 1.88] |  | 0.59 [0.46, 0.75] | 1.44 [1.21, 1.67] |  | -0.32 [-0.49, -0.16] |
| *Decision authority n=2865* |  | 0.0077 |  |  | <0.001 |  |
| Low | 2.34 [1.89, 2.89] |  | 0.62 [0.53, 0.74] | 2.00 [1.73, 2.26]^1)^ |  | -0.58 [-0.78, -0.39] |
| Middle | 1.77 [1.46, 2.13] |  | 0.61 [0.53, 0.69] | 1.62 [1.41, 1.82] |  | -0.42 [-0.53, -0.32] |
| High | 1.18 [0.94, 1.49] |  | 0.90 [0.72, 1.13] | 1.27 [1.07, 1.47] |  | -0.03 [-0.18, -0.12] |
| *Job strain n=2859* |  | 0.037 |  |  | <0.001 |  |
| No | 1.56 [1.31, 1.87] |  | 0.70 [0.63, 0.78] | 1.41 [1.23, 1.58] |  | -0.25 [-0.33, -0.16] |
| Yes | 2.47 [2.00, 3.05] |  | 0.55 [0.46, 0.67] | 2.04 [1.78, 2.30] |  | -0.68 [-0.92, -0.45] |
| ***B. Social living environment*** |  |  |  |  |  |  |
| *Neighborhood socioeconomic disadvantage n=2861* |  | 0.63 |  |  | 0.66 |  |
| Low | 1.67 [1.38, 2.02] |  | 0.61 [0.53, 0.72] | 1.45 [1.24, 1.65] |  | -0.34 [-0.46, -0.22] |
| Middle | 1.87 [1.55, 2.25] |  | 0.68 [0.59, 0.78] | 1.55 [1.35, 1.74] |  | -0.30 [-0.43, -0.17] |
| High | 2.09 [1.65, 2.64] |  | 0.65 [0.52, 0.80] | 1.72 [1.44, 2.00] |  | -0.42 [-0.67, -0.18] |
| *Neighborhood social cohesion n=3060* |  | 0.65 |  |  | 0.012 |  |
| Low | 2.98 [2.39, 3.71] |  | 0.66 [0.53, 0.82] | 2.47 [2.12, 2.82]^1)^ |  | -0.68 [-1.01, -0.36] |
| Middle | 1.92 [1.62, 2.28] |  | 0.62 [0.54, 0.70] | 1.72 [1.53, 1.92] |  | -0.44 [-0.56, -0.33] |
| High | 1.40 [1.15, 1.69] |  | 0.68 [0.58, 0.79] | 1.41 [1.21, 1.60] |  | -0.24 [-0.34, -0.13] |
| *Marital status n=3006* |  | 0.40 |  |  | 0.51 |  |
| Married or  cohabiting | 1.65 [1.40, 1.95] |  | 0.63 [0.56, 0.70] | 1.48 [1.30, 1.66] |  | -0.33 [-0.42, -0.24] |
| Living alone | 2.15 [1.77, 2.62] |  | 0.68 [0.58, 0.79] | 1.78 [1.55, 2.00] |  | -0.39 [-0.56, -0.23] |
| *Social network size n=3062* |  | 0.35 |  |  | 0.028 |  |
| Small | 2.74 [2.21, 3.39] |  | 0.62 [0.50, 0.76] | 2.28 [1.94, 2.62]^2)^ |  | -0.76 [-1.03, -0.49] |
| Middle | 1.83 [1.52, 2.21] |  | 0.69 [0.60, 0.80] | 1.52 [1.31, 1.73] |  | -0.35 [-0.48, -0.23] |
| Large | 1.51 [1.25, 1.81] |  | 0.60 [0.52, 0.70] | 1.33 [1.12, 1.53] |  | -0.39 [-0.50, -0.29] |
| ***C. Cumulative risk factors*** |  |  |  |  |  |  |
| *Psychosocial working conditions n=2865* |  | 0.46 |  |  | 0.001 |  |
| 0 | 1.42 [1.17, 1.73] |  | 0.70 [0.61, 0.81] | 1.20 [0.99, 1.40]^2)^ |  | -0.26 [-0.36, -0.16] |
| 1 | 1.82 [1.47, 2.24] |  | 0.63 [0.52, 0.76] | 1.52 [1.27, 1.76] |  | -0.47 [-0.64, -0.30] |
| ≥2 | 2.44 [1.99, 3.01] |  | 0.62 [0.53, 0.73] | 2.00 [1.72, 2.29] |  | -0.66 [-0.85, -0.46] |
| *Social living environment n=3073* |  | 0.74 |  |  | 0.060 |  |
| 0 | 1.49 [1.25, 1.78] |  | 0.62 [0.54, 0.72] | 1.36 [1.19, 1.53] |  | -0.29 [-0.39, -0.19] |
| 1 | 1.83 [1.51, 2.22] |  | 0.68 [0.58, 0.78] | 1.52 [1.33, 1.72] |  | -0.33 [-0.46, -0.20] |
| ≥2 | 2.93 [2.39, 3.61] |  | 0.64 [0.53, 0.77] | 2.32 [2.01, 2.62] |  | -0.66 [-0.96, -0.37] |
| *Psychosocial working conditions and social living environment n=3048* |  | 0.83 |  |  | <0.001 |  |
| 0 | 1.41 [1.15, 1.73] |  | 0.66 [0.54, 0.80] | 1.20 [1.00, 1.41]^2)^ |  | -0.29 [-0.42, -0.16] |
| 1 | 1.53 [1.25, 1.88] |  | 0.70 [0.59, 0.83] | 1.28 [1.07, 1.49] |  | -0.25 [-0.37, -0.12] |
| 2 | 1.97 [1.59, 2.44] |  | 0.62 [0.51, 0.77] | 1.63 [1.37, 1.88] |  | -0.50 [-0.69, -0.30] |
| 3 | 2.61 [2.06, 3.31] |  | 0.60 [0.47, 0.76] | 1.97 [1.63, 2.31] |  | -0.65 [-0.91, -0.39] |
| ≥4 | 3.39 [2.66, 4.20] |  | 0.64 [0.51, 0.81] | 2.69 [2.26, 3.12] |  | -0.92 [-1.28, -0.56] |

^1)^ Model run with poisson distribution to achieve convergence

^2)^ Model run with poisson distribution and without sleep difficulties to achieve convergence
